# Flow Cytometry as a Diagnostic Tool in the Early Diagnosis of Aggressive Lymphomas Mimicking Life-Threatening Infection

**DOI:** 10.1155/2011/743817

**Published:** 2011-06-05

**Authors:** Nikolaos J. Tsagarakis, Nektaria A. Kentrou, Georgios Kakiopoulos, Georgios Androutsos, Athanasios Galanopoulos, Christos Michaelidis, Dimitra Rontogianni, Apostolos Tolis, Stavroula Chini, Georgios Gortzolidis, Konstantinos A. Papadimitriou, Dimitra Skoumi, Konstantina Tzanetou, Georgios Paterakis

**Affiliations:** ^1^Flow Cytometry Laboratory, Department of Immunology, Athens Regional General Hospital “G. Gennimatas”, 11527 Athens, Greece; ^2^Department of Pathology, Athens Regional General Hospital “G. Gennimatas”, 11527 Athens, Greece; ^3^Department of Laboratory Hematology, Athens Regional General Hospital “G. Gennimatas”, 11527 Athens, Greece; ^4^Department of Clinical Hematology, Athens Regional General Hospital “G. Gennimatas”, 11527 Athens, Greece; ^5^1st Department of Internal Medicine, Athens Regional General Hospital “G. Gennimatas”, 11527 Athens, Greece; ^6^Department of Pathology, Evangelismos General Hospital, 10676 Athens, Greece; ^7^2nd Department of Internal Medicine, Athens Regional General Hospital “G. Gennimatas”, 11527 Athens, Greece; ^8^3rd Department of Internal Medicine, Athens Regional General Hospital “G. Gennimatas”, 11527 Athens, Greece; ^9^Department of Microbiology, Athens Regional General Hospital “G. Gennimatas”, Mesogion Avenue 154, 11527 Athens, Greece

## Abstract

Aggressive lymphomas can present with symptoms mimicking life-threatening infection. Flow cytometry (FC) is usually recommended for the classification and staging of lymphomas in patients with organomegaly and atypical cells in effusions and blood, after the exclusion of other possible diagnoses. FC may also have a place in the initial diagnostic investigation of aggressive lymphoma. Three cases are presented here of highly aggressive lymphomas in young adults, which presented with the clinical picture of fever of unknown origin (FUO) in patients severely ill. All followed a life-threatening clinical course, and two developed the hemophagocytic syndrome (HPS), but microbiological, immunological, and morphological evaluation and immunohistochemistry (IHC) failed to substantiate an early diagnosis. FC was the technique that provided conclusive diagnostic evidence of lymphoma, subsequently verified by IHC. Our experience with these three cases highlights the potential role of FC as an adjunct methodology in the initial assessment of possible highly aggressive lymphoma presenting with the signs and symptoms of life-threatening infection, although the definitive diagnosis should be established by biopsy. In such cases, FC can contribute to the diagnosis of lymphoma, independently of the presence of HPS.

## 1. Introduction

The clinical indications for flow cytometry (FC) are changing with the documentation of new evidence. The Bethesda 2006 Consensus decided on a list of clinical indications for FC [1], which include staging of disease, prognostic and therapeutic purposes, and monitoring of disease progress, but the use of FC in the initial assessment of life-threatening clinical situations, such as highly aggressive lymphoma, has not been evaluated. Lymphomas are seriously considered in the differential diagnosis of patients presenting with persistent fever of unknown origin (FUO) [2, 3], although the exclusion of an infectious cause is of major priority. The hemophagocytic syndrome (HPS), or hemophagocytic lymphohistiocytosis (HLH), can be associated with a variety of infections, autoimmune diseases, and congenital disorders, but also with lymphomas [4]. In cases of life-threatening infection with or without HPS, FC could possibly have an adjuvant diagnostic role in the initial assessment. 

In this paper, we present three cases which ultimately proved to be aggressive lymphomas, but all three initially presented as life-threatening infections with no evidence of an infectious causative agent, and two were associated with HPS. Our main purpose is to highlight the role of FC as an initial diagnostic tool in the assessment of such cases, in which a definitive diagnosis is urgently required. FC can provide strong evidence of lymphoma, but the final diagnosis should always be established by biopsy.

## 2. Case Series Presentation

### 2.1. Case  1

#### 2.1.1. Clinical Course

A 34-year-old male was admitted for the investigation of a 4-day fever and fatigue, accompanied by painful right inguinal lymphadenopathy and hepatosplenomegaly. During the first 2 weeks of hospitalization, the patient's condition deteriorated (Table 1), with the development of pleural effusion and hepatic and renal failure. The diagnosis of an infectious disease was strongly suspected, but cultures (from urine, blood, sputum, stool, and lymph node) and extensive investigation for viruses and other infectious agents were negative (Table 1). There was no clinical response to the administration of multiple antibiotics. FC analysis of the peripheral blood 20 days after admission, following the observation of atypical cells, was suspicious for a T-anaplastic lymphoma variant, and lymph node biopsy confirmed the FC findings. The Karyotype was normal and TCR rearrangements were negative.

#### 2.1.2. Morphology

Morphological examination of peripheral blood smear detected 30% atypical lymphocytes with irregular, eccentric, kidney-shaped, and inclusion-like nuclei, but also 2-3% rare large cells, with dispersed nuclear chromatin and basophilic microvacuolated cytoplasm,. The lymph node biopsy showed 20–30% large- and medium-sized cells with nucleoli and basophilic cytoplasm with small vacuoles. The other lymphocytes were small, with mature chromatin.

#### 2.1.3. FC

Peripheral blood FC showed similar findings to the lymph node FC analysis, on which the final diagnosis was based (Table 2). An atypical lymphocytic population of T-origin was detected, cCD3 brightly positive, CD2 positive, but negative for CD3, Tdt, TCR*αβ*, TCR*γδ* and CD5. Antigens of B and NK origin were absent, and Ki-67, as a marker of proliferation rate, was increased. CD25 was negative (regarding the possibility of an adult T-cell/leukemia/lymphoma), while the expression of CD30 and EMA, with concurrent absence of CD15, suggested a T-anaplastic non-Hodgkin lymphoma (NHL), Ki-1(CD30)+, small cell variant, which rarely presents with leukemic picture and with large, vacuolated, basophilic cells. 

#### 2.1.4. Immunohistochemistry (IHC)

IHC examination of bone marrow (BM) aspirate was not diagnostic, but lymph node processing revealed the diagnosis of a malignant diffuse NHL of T-origin (CD2+, UCHL-1+), with small- and medium-sized atypical cells, with CD30+, suggesting a T-anaplastic CD30+ lymphoma, small cell variant. 

#### 2.1.5. Outcome

Although treatment was delayed because of the delay in diagnosis, a complete remission was achieved.

### 2.2. Case  2

#### 2.2.1. Clinical Course

A 47-year-old male was admitted for the investigation of a 40-day FUO, with fatigue, weight loss, anemia, and thrombocytopenia. He had been hospitalized in another hospital, where no diagnosis was documented. His past medical history included chronic hepatitis B-virus infection, and heterozygosity for *β*-thalassemia. During hospitalization, the persistence of the fever and the development of a pericardial effusion and acute renal and respiratory failure indicated an infectious etiology. *A. baumannii* and *S. epidermidis* were isolated in blood cultures, but an extensive microbiological and immunological workup revealed no causative infectious agent (Table 1), and strong suspicion of histoplasmosis or parasitic infection (such as malaria and trypanosomiasis) was never confirmed. Antifungal treatment was administered, with no clinical response. New clinical signs appeared, including palpable lymph nodes, mild hepatosplenomegaly, hematuria, and bruises on the abdomen. The patient developed acute respiratory distress syndrome (ARDS) and was transferred to the Intensive Care Unit (ICU), where he died of acute pulmonary edema.

#### 2.2.2. Morphology

Almost 3% atypical cells were detected in blood and bone marrow (large- and medium-sized cells with nucleoli and basophilic, vacuolated cytoplasm), with hemophagocytosis.

#### 2.2.3. FC

**Figure 1:**
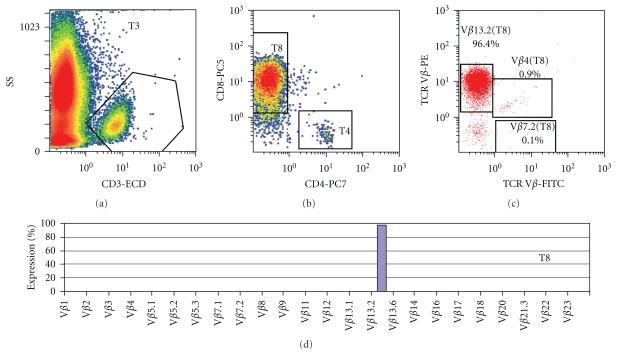
Flow cytometry of the bone marrow and peripheral blood of patient 2, showing a monoclonal TCR V*β*13.2 T-cell population, CD3+, TCR*αβ*+, CD8+, CD7+, CD30+, EMA+, ALK−, of high mitotic index (Ki-67 = 40%).

Immunophenotypic analysis of bone marrow aspirate detected a T-cell population, CD3+, TCRab+, CD8+, CD7+, brightly positive for cCD3 but CD10 and Tdt negative. It revealed a *restricted* expansion of TCRVb13.2 region, but was also highly mitotic (Ki-67 = 40%), ki-1/CD30+, EMA+, and ALK- (Figure 1). B- and NK- markers were negative (Table 2). 

#### 2.2.4. IHC

IHC and immunocytochemical analysis of the first bone marrow biopsy was inadequate to provide a conclusion, despite the presence of histiocytes and hemophagocytosis. The analysis of a second bone marrow aspirate documented infiltration (6-7%) by a peripheral highly aggressive T-NHL, CD8+, CD30+ population. 

#### 2.2.5. Outcome

The patient died shortly after the diagnosis was made on IHC.

### 2.3. Case  3

#### 2.3.1. Clinical Course

A 26-year-old female was admitted with a febrile disease of 40-day duration, with increasing fatigue and development of swellings all over her body. Hepatosplenomegaly and lymphadenopathy, small pericardial and pleural effusions, and renal failure and hepatic failure were observed, and anemia, thrombocytopenia, and increased C-reactive protein (CRP) were detected, all indicative of an infectious etiology. There was a strong suspicion of human zoonoses, but the microbiological and immunological investigation was inconclusive (Table 1). FC from a lymph node processed at the time of admission provided diagnostic evidence of HL. Lymph node IHC later verified the FC findings.

#### 2.3.2. Morphology

The processing of a lymph node section revealed a small number of Hodgkin-like cells, among small- and medium-sized lymphocytes with mature chromatin. The presence of monocytes with rare bilobed morphology, was supportive of a CD30+ anaplastic lymphoma, while the BM smear revealed hemophagocytosis. The morphological evaluation could not confirm Reed-Sternberg (RS) morphology.

#### 2.3.3. FC

**Figure 2:**
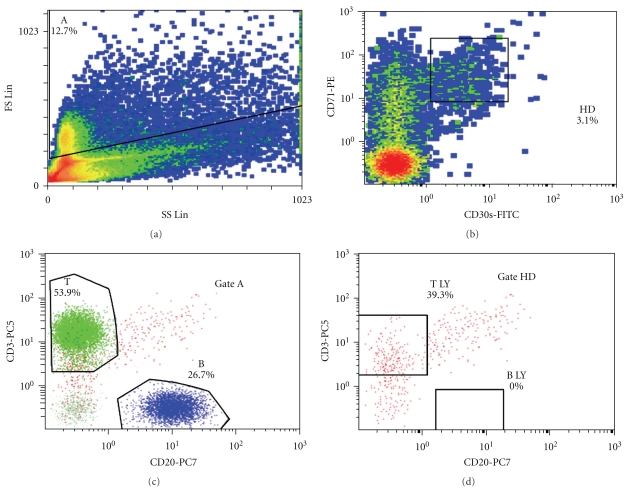
Flow cytometry of sample from the lymph node of patient 3. The coexpression of CD30 and CD71, in a CD45 and CD3 positive population of high forward scatter characteristics, indicated the possibility of Hodgkin and Reed Sternberg (HRS) cells, based on T cell rosetting around HRS cells. This composite immunophenotype (CD45+/CD30+/CD71+/CD3+/CD20−) (gate HD) was considered due to contributions by the HRS cells and the surrounding T cells.

**Figure 3:**
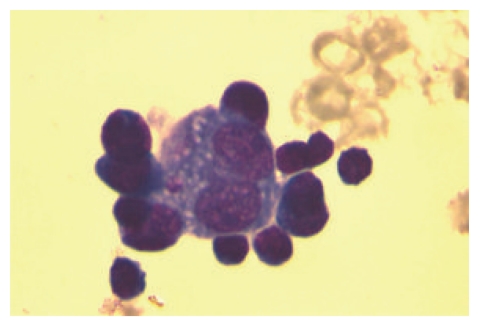
Microscopy of pleural effusion specimen from patient 3 showing Hodgkin-like cells, surrounded by atypical lymphocytes, probably T-cells (HRS-cell-T-cell rosette).

Lymph node tissue section and pleural effusion were analyzed by FC. In the lymph node, the lymphocytes (90% of total cells) were mature cells, including 55% T (CD3+) and 33% B (CD19+). There was a predominant T4 population, but also T8 cells and polyclonal B-cells were detected. A nonhematolymphoid neoplastic cell population was excluded in the pleural effusion (cytokeratin, EMA, and BerEP4 negative). Classical HL was suggested by the lymph node findings, because of the composite immunophenotype CD45+/CD30+/CD71+/CD3+/CD20−, in a high forward-scattered cell population (Figure 2), with CD15dim expression. The FC conclusions were based also on the morphological suspicion of Hodgkin and RS (HRS) cells, particularly in the pleural effusion (Figure 3). TCR*γδ* lymphocytes were not detected in the lymph node, so the possibility of a hepatosplenic TCR*γδ*-lymphoma was eliminated. 

#### 2.3.4. IHC

IHC processing of an infiltrated axillary lymph node provided the diagnosis of classical HL, although there had been strong suspicion of NHL, because of certain features overlapping with those of T-anaplastic large cell lymphoma (ALCL), or angioimmunoblastic T-cell lymphoma (AITL). Such findings were the growth pattern (sinusoidal), the hemophagocytic syndrome, the abundance of malignant cells, and the difficulty in classifying the entity into an HL subtype (“grey-zone” lymphoma). The IHC diagnosis was based on the low coexpression of CD15 with CD30 in neoplastic cells, together with the findings of Pax-5+, MUM-1+, Oct-2−, LCA−, EMA−, CD20−, CD4−, CD56−, and Granzyme-B−. Investigation for ALK protein and Epstein-Barr (EB) protein (LMP-1) was negative. 

#### 2.3.5. Outcome

Bleo-CHOP therapy was started two days after admission, as there was no certain diagnosis of HL apart from the FC findings. This was followed by the ABVD regimen, based on the definitive diagnosis of classical HL established by IHC. A complete remission was achieved initially, followed by relapse and death 6 months later.

## 3. Materials and Methods

### 3.1. FC

FC was performed on cell suspensions from peripheral blood, bone marrow aspirate, or fresh lymph node tissue using previously described methods [5]. Briefly, after a 15-minute incubation of a suspension of cells labeled with fluorescent antibodies (10 *μ*L, undiluted) at room temperature, cells were thoroughly lysed (VersaLyse Lysing Solution, OITest-3 Fixative Solution, Beckman Coulter) for 10 minutes and centrifuged (1800 rpm, 5 minutes). The supernatant liquid was discarded, and the cells were resuspended in 0.5ml of PBS for analysis. For combined cell surface (s) and cytoplasmic (c) immunophenotyping, cells were incubated for 15 minutes with surface-reactive labeled antibodies, followed by a second 15-minute incubation with 100 *μ*L Fix & Perm reagent “A” and then washed once. After the addition of 100 *μ*L Fix & Perm reagent “B”, cells were incubated for 15 minutes with antibodies to cytoplasmic antigens, washed, and analyzed on the cytometer. The Fix & Perm reagent kit (Caltag Laboratories, San Francisco, CA, USA) was used for cell fixation and permeabilization. Tissue specimens were minced in 3-4 mL of PBS using a scalpel. Tissue homogenates were used for touch imprints, which were stained with May-Grunwald/Giemsa, followed by the preparation of single-cell suspensions by filtering through 100-*μ*m filters. FC analysis was performed on a 5-color flow cytometer, FC500 (Beckman Coulter). Analysis was made with CXP software (Beckman Coulter's), and at least 20,000 events were counted for each sample.

The fluorochrome combinations used included fluorescein isothiocyanate (FITC), phycoerythrin (PE), PE-Texas red (ECD), PE-cyanine (Cy)-5 (PC5), and PE-Cy7 (PC7). The fluorescently labeled antibodies were obtained from Beckman Coulter and DakoCytomation, and included IgG1-FITC, IgG1-PE, CD3-FITC/PE/ECD/PC5, CD19-PC7, CD20-PE/ECD/PC7, CD45-ECD/PC5, kappa-FITC, lambda-PE, Tdt-FITC, bcl2-FITC, CD5-FITC/PC5, CD79a-PE, CD38-FITC/PC5, BB4-PE, CD10-PE, EMA-FITC, CD30(Ki-1)-FITC, CD15-FITC, HLA-DR-PE, Ki-67-FITC, IgM-FITC, CD4-PE/PC7, CD8-FITC/PC5, CD2-FITC, CD7-FITC, CD1a-FITC, CD14-FITC/PC7, ALK-FITC, CD16-FITC/PE/PC5, CD56-PE/PC7, CD71-PE, TCR*αβ*-PE, TCR*γδ*-FITC, CD25-PE, cytokeratin-FITC, CD45RO-FITC, and CD45RA-PE. Appropriate isotype controls were used. FC analysis of TCR-V_*β*_ expression was performed with the IOTest Beta Mark TCR-V_*β*_ Repertoire kit (Beckman Coulter, Miami, FL), as previously described [6]. For investigating the possibility of HL (in Case  3), cells with high forward scatter characteristics, which coexpressed CD71 and CD30, were examined for B-(CD20) and T-cell markers (CD3), based on the observation of T-cell rosetting around HRS cells, as previously proposed [7]. The final report was always combined with morphology and clinical features. 

### 3.2. IHC and Immunocytochemical Analysis

Bone marrow biopsies and lymph node tissue sections were studied by IHC and immunocytochemistry. Immunostaining was carried out in 4-*μ*m formalin-fixed and paraffin embedded sections. Among the primary antibodies used were L26, CD79a, CD3, CD2, UCHL1 (T cells/CD45RO), MT1 (T cells/CD43), Anti-kappa, Anti-lambda, BerH2 (activated lymphocytes, Hodgkin cells/CD30), LeuM1 (Hodgkin cells/CD15), CD5, CD10, EMA, ALK, BOB1, Pax-5, Oct2, CD34, bcl2, bcl6, CD23, ki-67, and KP1 (histiocytes/CD68), using an automated Envision/HPR technique (DAKO cytomation, Denmark). The final diagnosis was made by two independent observers, after the evaluation of morphological and ICH findings in combination with the clinical data, without knowledge of the FC results.

## 4. Discussion

Consensus has been reached on a list of clinical indications for FC analysis [1], including patients with hepatosplenomegaly and lymphadenopathy and/or atypical cells in the blood or pleural effusions, but the recommendations are to first rule out other possible causes of the clinical signs and symptoms. There has been no evaluation of the utility of FC in the initial investigation for life-threatening conditions, such as aggressive lymphomas, in severely ill patients with the clinical picture of FUO. In order to highlight the potential role of FC analysis in the critical care situation, we have described three cases of lymphoma presenting as acute infection, two of which were associated with HPS. The aggressive clinical course of these cases demanded a rapid initial assessment, in order to determine the diagnosis and initiate appropriate treatment to try to ensure the survival of the patients. All three cases were thoroughly investigated for an underlying infection or autoimmune disease with negative results, and FC was the only laboratory method to provide early diagnostic evidence of lymphoma. The definitive diagnosis, obtained later by IHC, confirmed the initial FC findings. One patient (Case  2) died untreated, while the other two patients (Cases  1 and 3) achieved remission, despite critical delay in diagnosis. 

All three cases presented as FUO, while Cases  2 and 3 also showed HPS. In cases of HPS and FUO, lymphoma entities should be seriously considered in the differential diagnosis. Several cases of B- and T-cell lymphoma associated with HPS have been documented [8–16]. Tong et al. [17] have suggested that the median survival of patients with lymphoma associated with HPS was much lower than that of those with non-HPS associated lymphoma. It is apparent that delay in the time-consuming procedure of the differential diagnosis between infection, HPS-associated infection, and HPS-associated lymphoma can significantly increase the risk of a fatal outcome. 

FC provided early diagnostic evidence in all three cases, although the initiation of appropriate treatment was delayed, pending the IHC confirmation. In Case  1, the late immunophenotyping of peripheral blood revealed the expression of CD30 and EMA, with concurrent absence of CD15, suggesting a Ki-1 anaplastic large-cell lymphoma, which rarely presents with a leukemic picture and with large, vacuolated basophilic cells. The awareness of a small-cell variant [18] led to the study of a lymph node tissue section, where the immunophenotype was compatible with T-anaplastic lymphoma. FC investigation of CD25 expression excluded the possibility of adult T-cell/leukemia/lymphoma, and lymph node processing by IHC later confirmed FC results. In Case  2, the FC diagnosis was that of a T-anaplastic lymphoma, Ki-1 (CD30) positive, ALK negative. The second, diagnostic, bone marrow biopsy documented the infiltration by a highly aggressive peripheral T-NHL, CD8+, CD30+. Unfortunately, the diagnosis came too late as the patient died shortly after the histological report. In Case  3, although IHC provided the final diagnosis of HL, the possibility of a “grey-zone” lymphoma had been previously discussed. FC had earlier raised suspicions of HL, based on the morphological features of HRS cells in the pleural fluid, coupled with the touch imprints. 

Despite the limited documentation of the contribution of FC to the diagnosis of HL, and in the absence of a standardized, well-accepted protocol, we based our FC approach on the recently published paper by Fromm et al. [7, 19]. In their efforts to identify HRS cells in lymph nodes, FC assay revealed diagnostic sensitivity and specificity of 88.7% and 100%, respectively [19]. HRS cells are often bound to T-cells, typically showing subsets of HRS cells with and without attached T cells (i.e., with or without rosette formation). HRS-cell-T-cell rosettes had a composite immunophenotype (with antigen contribution from the T cells and the HRS cells) when characterized by FC [7]. These investigations were made with 10- [7] or 9-colour [19] flow cytometric assays, but with only 5-colour availability, we had to modify our approach, which was based on the concept of HRS-cell-T-cell rosette formation. The high forward scatter characteristics and the T-cell immunophenotypic contribution of CD3 and CD45 bright expression, in association with CD20 absence, in a CD30/CD71 positive, CD15dim population was strongly supportive of HRS cells. The morphological study of pleural effusion supported our approach (Figure 3).

The initial morphological and ICH diagnostic speculations in Case  3 were partially based on familiar morphological and immunophenotypic overlapping features. A morphological and immunophenotypic overlap of ALCL with classic HL has been recognized [20]. The expression of ALK fusion proteins (chimeric ALK) has minimized the difficulty in differentiation between ALCL and HL in cases that lack expression of T-cell and B-cell antigens, but there is still an overlap between ALK-negative ALCL and HL (e.g., CD15+/CD30+). It is clear that consensus histological and IHC criteria for the diagnosis of ALK(−) ALCL are lacking [21]. With regard to CD30 expression, the HRS cells of HL and the neoplastic cells of ALCL both express CD30 [22], which does not, however, have disease specificity, as it is an activation-associated antigen [23–27].

Concisely, the cases we have presented concerned unusual types of lymphoma, with rare clinical and laboratory manifestations, mimicking FUO in patients severely ill but also associated with secondary hemophagocytosis. Their highly aggressive clinical course demanded a rapid and safe diagnostic approach, to try to ensure survival, and in this context, FC proved to provide a rapid diagnostic assessment. Undoubtedly, IHC is the essential and most reliable diagnostic tool and remains the gold standard of lymphoma diagnosis; however, it is a tool which may involve difficulties, related to inadequate samples, differential diagnostic problems, or critical delays, before the definitive diagnosis is reached. Through the 3 cases described, we have tried to highlight the potential of FC, combined with morphology, as a valuable informative tool for the early assessment of life-threatening conditions. We recommend that it should be considered as an initial test in all undiagnosed cases of FUO with organomegaly and/or atypical cells in blood or pleural effusion, in order to assess the possibility of an underlying hematolymphoid neoplasm.

## Figures and Tables

**Table 1:** The main clinical features and laboratory results.

	Case 1	Case 2	Case 3
Clinical features			
Fever	+	+	+
Hepatosplenomegaly	+	+	+
Lymphadenopathy	+	+	+
Weakness	+	+	+
Weight loss	−	+	−
DIC signs	−	−	+
Swellings	−	−	+
Pleural effusion	+	**+**	+
Hepatic failure	+	−	+
Renal failure	+	+	+

Laboratory findings			
hemoglobin (Hb)	↓	↓	↓
platelets (PLTs)	↓	↓	↓
White blood cells (WBCs)	↑	↑	↓
C-reactive protein (CRP)	↑	**↑**	**↑**
Erythrocyte sedimentation rate (ESR)	↑	**↑**	~
Lactate dehydrogenase (LDH)	↑	**↑**	**↑**
*β*_2_-microglobulin	↑	**↑**	**↑**
Ferritin	↑	**↑**	**↑**
d-dimers	−	**↑**	~
CMV, EBV, Parvo-B19, herpes viruses and Coxsackie B1-B6	−	−	−
Hepatitis-B virus (HBV)	−	+	−
Hepatitis-C virus (HCV)	−	−	−
Human immunodeficiency virus (HIV)	−	−	−
Human T-lymphotropic viruses (HTLV-I, -II)	−	−	−
Syphilis (VDRL)	−	−	−
*Brucella *	−	−	N
*Toxoplasma *	−	−	−
*Chlamydia pn. *	−	−	N
*Yersinia, Listeria *	−	N	−
*Leptospira *	−	N	−
*Legionella, Pneumococcus urine antigens *	N	−	N
*Bartonella *	−	N	−
*Histoplasma *	N	−	N
*Malaria *	N	−	N
*Leishmania *	N	−	−
*Other parasitic infections *	−	N	−
*Rickettsiae *	N	−	−
*M. tuberculosis *	−	N	−
CSF	−	N	N

↑: increase, ↓: decrease, +: positive, ~: not stable, NS: no significant alteration, N: not evaluated, DIC: disseminated intravascular coagulation, VDRL: Venereal Disease Research Laboratory test.

**Table 2:** Immunophenotype of atypical cell populations.

	Case 1		Case 2
	PB	LN	BM, PB
Atypical lymphocytes	T (31%*)	T (30.5%*)	T8 (3%*)
CD3	1%	1%	100%
cCD3	100%	100%	100%
CD4	100%	100%	0%
CD8	1%	1%	100%
CD2	100%	N	5%
CD7	35%	54%	95%
CD5	9%	8%	3%
TCR*αβ*	0.4%	0.5%	100%
TCR*γδ*	0.5%	0.5%	0%
Tdt	1%	N	0%
CD34	1%	N	0%
CD30(Ki-1)	55%	79.5%	41%
EMA	47%	30%	80%
CD15	3%	6%	2%
ALK	N	N	0%
CD10	0%	N	0%
CD19	0%	N	0%
CD20	0%	N	0%
CD16	1.3%	N	0%
CD56	1%	N	0%
HLA-DR	78%	N	N
CD25	5%	15%	N
CD14	0%	N	0%
Ki-67	3%	22.6%	40%
CD45R0	100%	N	N
CD45RA	0.7%	N	N

PB: peripheral blood, BM: bone marrow, LN: lymph node, c: cytoplasmic, N: not evaluated, *(%) of total nucleated cells, while all other percentages refer to the atypical cell populations.
